# Association of Appendicular Skeletal Muscle Mass Index and Insulin Resistance With Mortality in Multi‐Nationwide Cohorts

**DOI:** 10.1002/jcsm.13811

**Published:** 2025-04-14

**Authors:** Shinje Moon, Jong Wook Choi, Jung Hwan Park, Dong Sun Kim, Youhern Ahn, Yeongmin Kim, Sung Hye Kong, Chang‐Myung Oh

**Affiliations:** ^1^ Department of Internal Medicine Hanyang University College of Medicine Seoul South Korea; ^2^ Department of Biomedical Science and Engineering Gwangju Institute of Science and Technology Gwangju South Korea; ^3^ Department of Internal Medicine Seoul National University Bundang Hospital Seongnam South Korea

**Keywords:** all‐cause mortality, cardiovascular disease, insulin resistance, metabolic syndrome, sarcopenia

## Abstract

**Background:**

Although sarcopenia and insulin resistance are closely related, there is limited evidence regarding how they interact to influence mortality across different population groups. The purpose of this study was to examine the relationship between skeletal muscle mass and insulin resistance and its impact on mortality and cardiovascular disease risk using large‐scale national data from Korea and the United States.

**Methods:**

We analysed data from the National Health and Nutrition Examination Survey (NHANES) 1999–2006 and 2011–2018 and the Korea National Health and Nutrition Examination Survey (KNHANES) 2008–2011, with mortality follow‐up through to 2019. Cox regression models were used to assess the effects of muscle mass (appendicular skeletal mass index, ASMI) and insulin resistance on all‐cause and major adverse cardiovascular and cerebrovascular events (MACCE)–related mortality. Mediation analysis was performed to examine direct and indirect effects.

**Results:**

The study included 8036 participants from NHANES and 14 449 from KNHANES. The sarcopenia group demonstrated a lower homeostasis model assessment for insulin resistance and better metabolic indices than the normal group despite having a higher mortality rate. Insulin resistance positively correlated with muscle mass (*r* = 0.203, *p* < 0.001 in the NHANES; *r* = 0.143, *p* < 0.001 in the KNHANES), and both insulin resistance and sarcopenia were identified as independent risk factors for all‐cause and MACCE‐related mortality. When the participants were categorized into four groups based on the presence or absence of insulin resistance and sarcopenia, those with both conditions exhibited the highest risk of all‐cause mortality (hazard ratio [HR]: 2.30, 95% confidence interval [CI]: 1.72–3.08 in the NHANES; HR: 2.60, 95% CI: 2.14–3.16 in the KNHANES) and MACCE‐related mortality among the groups (HR: 3.18, 95% CI: 1.99–5.08 in the NHANES; HR: 2.47, 95% CI: 1.66–3.69 in the KNHANES). Mediation analysis revealed that low muscle mass was associated with decreased insulin resistance but directly increased both all‐cause mortality and MACCE‐related mortality (NHANES: total natural direct effects [TNDE], HR: 2.08, 95% CI: 1.57–2.76; KNHANES: TNDE, HR: 1.69, 95% CI: 1.28–2.23).

**Conclusions:**

This study found that low ASMI was inversely associated with insulin resistance and positively associated with mortality risk in both cohorts. These findings, consistent across two large national studies, highlight the complex relationships between muscle mass, insulin sensitivity and mortality. Further studies are needed to assess the underlying mechanisms and clinical implications of these associations.

**Trial Registration:**

Clinicaltrials.gov ID: NCT05616013

## 1 Introduction

Sarcopenia is characterized by a loss of muscle mass and an impairment of muscle strength or physical performance [1]. The definition of sarcopenia has evolved because of ongoing research on screening tools and the creation of simplified diagnostic algorithms and clinical guidelines by various international organizations. As awareness of this condition has increased, clinical diagnoses have also risen [2]. The prevalence of sarcopenia increases with age. According to the 2014 Asian Working Group for Sarcopenia criteria, the prevalence of sarcopenia among community‐dwelling older adults in Asian countries ranges from 5.5% to 25.7%. In Korea, the prevalence of sarcopenia ranges from 4% to 45% in older adults according to different definitions [3]. Globally, the prevalence of sarcopenia in older adults ranges from 5% to 17% [4]. As sarcopenia disproportionately affects older and vulnerable populations, its clinical relevance has rapidly increased [3]. Advanced age is a significant risk factor for both the onset and severity of sarcopenic obesity, which is characterized by an increase in visceral fat along with a decline in muscle mass and function. In the elderly population, the prevalence of sarcopenic obesity has reached 11%, with a notable increase observed in individuals over the age of 70 [5]. Sarcopenia is associated with several adverse health outcomes, including reduced physical function, increased frailty, chronic metabolic risk and mortality [6].

Insulin resistance (IR), a central feature of metabolic syndrome [7], is characterized by a dysregulated biological response to insulin, leading to increased glycemia and an increased risk of Type 2 diabetes (T2D), hypertension (HTN) and dyslipidaemia [8]. It is well known that IR is a significant predictor of cardiovascular disease (CVD) and all‐cause mortality [9]. In both non‐diabetic and diabetic populations, the Homeostasis Model Assessment for Insulin Resistance (HOMA‐IR) has shown positive associations with CVD and all‐cause mortality [9, 10, 11].

Emerging evidence suggests a significant interaction between sarcopenia and IR. Sarcopenia may contribute to the development of IR, which in turn increases the risk of metabolic disease and mortality [12, 13, 14]. On the other hand, IR may also play a critical role in the pathogenesis of induced muscle wasting [15, 16]. Although animal studies have firmly established an association between sarcopenia and IR [16, 17], the epidemiological evidence from human studies remains limited [18, 19]. Furthermore, the synergistic effects of low muscle mass and IR on mortality have not been extensively studied, particularly in diverse ethnic populations.

To address this knowledge gap, we conducted a comprehensive study using data from multi‐nationwide cohorts including the National Health and Nutrition Examination Survey (NHANES) [20] and the Korea National Health and Nutrition Examination Survey (KNHANES) [21]. Our primary objective was to examine the associations between sarcopenia and IR, and their combined effects on all‐cause and major adverse cardiovascular and cerebrovascular events (MACCE)–related mortality. To evaluate the complex interplay, we analysed the direct and indirect associations between low muscle mass and increased mortality risk according to IR.

## 2 Methods

### 2.1 Study Population

This study used data from the NHANES, a cross‐sectional survey that collects information on health, nutrition, medical, dental, physical measurements and laboratory analyses from a representative sample of the US population [22]. The researchers obtained baseline data from four NHANES cycles (1999–2006 and 2011–2018). To conduct a longitudinal study, the baseline data from these NHANES cycles were connected to mortality data from the National Death Index, which provides information on deaths up to 2019 [23].

This study also utilized data from the KNHANES, a cross‐sectional survey that is nationally representative of the noninstitutionalized civilian population in South Korea. The KNHANES is conducted by the Korean Centers for Disease Control and Prevention (KCDC). The researchers used KNHANES data collected between 2008 and 2011. The survey employed a multistage stratified sampling design to ensure representativeness. To perform the longitudinal analysis, the KNHANES data were linked to mortality data from the National Death Registry, maintained by the Korea National Statistical Office, which provided information on participant deaths up to 2019.

### 2.2 Measurements

In the NHANES, participant blood pressure (BP) was measured three times after a minimum rest of 5 min while sitting, and the mean of the three readings was taken. Enzymatic methods were used to measure fasting blood glucose and cholesterol levels. The fasting insulin concentrations were measured using the Pharmacia Insulin RIA kit (Pharmacia Diagnostics AB, Uppsala, Sweden) from 1999 to 2002, the Tosoh AIA‐PACK IRI (Tosoh Bioscience Inc., Grove City, OH, USA) from 2003 to 2004, the Merocodia Insulin ELISA (Merocodia, Uppsala, Sweden) from 2005 to 2006, the Elecsys 2010 analyser (Roche, Basel, Switzerland) from 2011 to 2012 and Tosoh Bioscience AIA‐900 (Tosoh Bioscience Inc., Grove City, OH, USA) from 2013 to 2018. The NHANES laboratory procedure manual provides additional information regarding sample collection and testing methodologies [24]. Whole‐body dual X‐ray absorptiometry (DXA) scans were performed using a Hologic QDR 4500A fan‐beam X‐ray bone densitometer (Hologic Inc., Marlborough, MA, USA) in NHANES 1999–2006 and Hologic Discovery Model A densitometers in NHANES 2011–2018. Total and regional body compositions were assessed using DXA scans.

In the KNHANES, BP was measured three times in the sitting position after at least 5 min of rest, and the mean of the three readings was calculated. Fasting blood glucose and cholesterol levels were measured enzymatically using a Hitachi Automatic Analyser 7600 (Hitachi Ltd., Tokyo, Japan). Fasting Insulin concentrations were measured with an immunoradiometric assay using the 1470 WIZARD automatic gamma counter (PerkinElmer, Turku, Finland). Whole‐body DXA scans were conducted using a QDR Discovery fan beam densitometer (Hologic Inc., Bedford, MA, USA), and total and regional body compositions were analysed using DXA scans. Additional details regarding sample collection and examination methods can be found in the KNHANES guidelines [25, 26].

### 2.3 Definitions of IR and Low Muscle Mass

IR was calculated as follows:

$$\begin{array}{c} \text{HOMA}-\mathrm{IR}=\text{fasting insulin}\;\left(\mathrm{mU}/\mathrm{mL}\right)\\ \;\times \text{fasting glucose}\;\left(\text{mmol}/L\right)/22.5. \end{array}$$

We defined IR as a HOMA‐IR value above the 75th percentile or the use of medications for diabetes [27, 28]. Appendicular skeletal mass was defined as the sum of the total lean mass, excluding the bone mineral content of both arms and legs. The appendicular skeletal mass index (ASMI) was defined as the value obtained by dividing the appendicular skeletal mass by the square of the height (m) [29]. Low muscle mass was defined as an ASMI of < 7 kg/m^2^ for men or < 5.5 kg/m^2^ for women according to the European Working Group on Sarcopenia in Older People 2 (EWGSOP2) [30] in the NHANES and an ASMI of < 7.0 kg/m^2^ for men and < 5.4 kg/m^2^ for women according to the Asian Working Group on Sarcopenia in the KNHANES [31]. We calculated participants' relative muscle mass using ASM and body weight (appendicular skeletal lean mass divided by body weight, ASM/body mass) in the NHANES cohort [32, 33]. We also evaluated another relative muscle mass index that adjusts muscle mass for fat mass: appendicular muscle mass divided by total body fat mass (aMFR) [34].

### 2.4 Study Outcomes

Data on all‐cause and MACCE‐related mortality and follow‐up duration by years were gathered from public‐use–linked mortality data at the National Center for Health Statistics for the NHANES and the National Death Registry of the Korea National Statistical Office for the KNHANES until 31 December 2019.

### 2.5 Definition of Covariates

Covariates included age, sex, race/ethnicity, smoking status, alcohol consumption, history of cancer, estimated glomerular filtration rate (eGFR), dyslipidaemia and HTN at baseline. The questionnaire collected information on age, sex, race/ethnicity, smoking status, alcohol intake and history of cancer. The eGFR was calculated using the CKD Epidemiology Collaboration (CKD‐EPI) creatinine equation. HTN was defined as a systolic BP of more than 140 mmHg, mean diastolic BP of more than 90 mmHg or treatment for HTN. Dyslipidaemia was defined as a fasting total cholesterol of 240 mg/dL or more or treatment for dyslipidaemia. Alcohol intake was measured by asking each participant to quantify the number of drinks consumed, and drinkers were considered if they consumed one or more drink per month.

### 2.6 Statistical Analysis

For basic demographic characteristics, underlying diseases, anthropometric indices and blood test results, continuous variables are presented as means ± standard deviations, whereas categorical variables are presented as counts and percentages. We employed multivariate Cox proportional hazards models to calculate hazard ratios (HRs) for all‐cause mortality and MACCE‐related deaths. These models were adjusted for potential confounders including demographic factors (age, sex and race), lifestyle behaviours (smoking status and alcohol consumption), clinical parameters (eGFR) and medical history (cancer, hypertension and dyslipidaemia). The study's observation period was defined as the interval between the initial collection of anthropometric and clinical data and either the participant's death or the study's end date (31 December 2019), whichever occurred first.

We employed causal mediation analysis using regression techniques to isolate the direct impact of low muscle mass on mortality, independent of metabolic health status. This analysis was performed using the ‘Regmedint’ package, a tool developed by Yoshida and colleagues [35] This R package serves as an equivalent to the mediation macro developed by Valeri and VanderWeele for SAS software [36, 37]. Our analysis encompassed various causal effects on mortality, including total and pure natural indirect effects (TNIE and PNIE), total and pure natural direct effects (TNDE and PNDE) and the overall total effect (TE). Statistical analyses were performed using two software packages: IBM SPSS Statistics version 24.0 (IBM Corporation, Armonk, NY, USA) and R version 4.2.0 (R Foundation for Statistical Computing, Vienna, Austria). The threshold for statistical significance was set at a *p*‐value of less than 0.05.

## 3 Results

### 3.1 Baseline Characteristics of the Participants

This study included 8036 participants from NHANES and 14 449 from KNHANES (Figure S1). Among the participants from the NHANES, 7052 had normal muscle mass and 984 had low muscle mass. The mean age of the participants with normal muscle mass was 45.1 years, and 48.0% were women, whereas the mean age of the participants with low muscle mass was 50.9 years (*p* < 0.001), and 58.7% were women (*p* < 0.001). Among the KNHANES participants, 10 952 had normal muscle mass and 3497 had low muscle mass. The mean age of participants with normal muscle mass was 48.3 years, and 55.4% were women; the mean age of participants with low muscle mass was 48.7 years (*p* = 0.231), and 60.6% were women (*p* < 0.001).

The demographic and clinical characteristics of the participants with and without low muscle mass in both the US and Korean cohorts are summarized in Table 1. In the NHANES, US adults with low muscle mass had a significantly lower HOMA‐IR (2.1 vs. 3.9, respectively; *p* < 0.001) than those with normal muscle mass. In addition, participants with low muscle mass had lower levels of fasting glucose, and triglycerides and higher levels of high‐density lipoprotein cholesterol than those with normal muscle mass. Participants with low muscle mass had a higher prevalence of malignancy (5.6% vs. 10.6%, respectively; *p* < 0.001), all‐cause mortality (11% vs. 29%, respectively; *p* < 0.001) and MACCE‐related mortality (3.3% vs. 12.2%, respectively; *p* < 0.001) than those with normal muscle mass.

**TABLE 1:** Overall characteristics of the participants according to muscle mass.

	NHANES			KNHANES		
	Normal (*N* = 7052)	Low muscle mass (*N* = 984)	*p*	Normal (*n* = 10 952)	Low muscle mass (*n* = 3497)	*p*
Age	45.1 ± 14.9	50.9 ± 19.2	< 0.001	48.3 ± 15.2	48.7 ± 17.6	0.231
Female sex, *n* (%)	3383 (48.0%)	578 (58.7%)	< 0.001	6150 (55.4%)	2179 (60.6%)	< 0.001
Race/ethnicity, *n*			< 0.001			
Hispanic	1802 (25.6%)	204 (20.7%)				
Non‐Hispanic White	3096 (43.9%)	541 (55.0%)				
Non‐Hispanic Black	1432 (20.3%)	51 (3.4%)				
Other races	722 (10.2%)	188 (19.1%)				
Smokers, *n* (%)	3121 (44.3%)	484 (49.2%)	0.004	4348 (39.3%)	1360 (37.9%)	0.130
Drinkers, *n* (%)	4930 (69.9%)	636 (64.6%)	< 0.001	6207 (56.0%)	1814 (50.5%)	0.147
BMI, kg/m^2^	29.2 ± 5.8	21.8 ± 2.7	< 0.001	24.5 ± 3.1	20.7 ± 2.2	< 0.001
ASMI, kg/m^2^	8.0 ± 1.5	5.7 ± 0.8	< 0.001	7.0 ± 1.1	5.6 ± 0.8	< 0.001
ASM/Total fat ratio	0.92 ± 0.43	0.92 ± 0.47	0.821	1.18 ± 0.60	1.13 ± 0.64	< 0.001
Systolic BP, mmHg	122.3 ± 17.7	123.3 ± 21.9	0.191	120.1 ± 17.2	116.5 ± 18.3	< 0.001
Diastolic BP, mmHg	71.8 ± 12.0	69.2 ± 12.4	< 0.001	77.7 ± 10.9	74.2 ± 10.4	< 0.001
Fasting glucose, mg/dL	107.4 ± 36.6	102.7 ± 31.1	< 0.001	98.0 ± 21.3	95.6 ± 24.5	< 0.001
Fasting insulin, μU/mL	13.5 ± 17.9	7.6 ± 5.6	< 0.001	10.2 ± 4.8	8.7 ± 3.9	< 0.001
HOMA‐IR	3.9 ± 6.7	2.1 ± 2.3	< 0.001	2.5 ± 1.7	2.1 ± 1.2	< 0.001
Insulin resistance, *n* (%)	1962 (27.8%)	104 (10.6%)	< 0.001	3314 (29.9%)	640 (17.8%)	< 0.001
Diabetes mellitus, *n* (%)	1027 (14.5%)	94 (9.6%)	< 0.001	1080 (9.7%)	322 (9.0%)	0.164
Total cholesterol, mg/dL	198.7 ± 43.4	198.5 ± 42.9	0.891	189.0 ± 36.0	182.7 ± 34.6	< 0.001
Triglycerides, mg/dL	143.9 ± 155.0	119.5 ± 91.1	< 0.001	138.7 ± 109.6	114.9 ± 95.9	< 0.001
HDL cholesterol, mg/dL	51.9 ± 15.0	59.7 ± 17.7	< 0.001	47.2 ± 10.6	50.6 ± 11.6	< 0.001
eGFR, mL/min/1.73 m^2^	99.1 ± 19.4	98.7 ± 23.0	0.574	99.8 ± 15.7	102.1 ± 17.9	< 0.001
Hypertension, *n* (%)	2825 (40.1%)	400 (40.7%)	0.723	3696 (33.3%)	910 (19.8%)	< 0.001
Dyslipidemia, *n* (%)	3014 (42.7%)	404 (41.1%)	317	1429 (12.9%)	321 (8.9%)	< 0.001
Malignancies, *n* (%)	392 (5.6%)	104 (10.6%)	< 0.001	288 (2.6%)	124 (3.4%)	0.007
All‐cause death, *n*	773 (11%)	285 (29%)	< 0.001	608 (5.5%)	437 (12.1%)	< 0.001
Noncancer‐related death, *n* (%)	581 (8.2%)	234 (23.8%)	< 0.001	398 (3.6%)	313 (8.7%)	< 0.001
Cancer‐related death, *n* (%)	192 (2.7%)	51 (5.2%)	< 0.001	210 (1.9%)	124 (3.4%)	< 0.001
MACCE‐related death, *n* (%)	231 (3.3%)	94 (12.2%)	< 0.001	142 (1.3%)	100 (2.8%)	< 0.001

Abbreviations: ASMI, appendicular skeletal muscle index; BMI, body mass index; BP, blood pressure; eGFR, estimated glomerular filtration rate; HDL, high‐density cholesterol; HOMA‐IR, The Homeostasis Model Assessment for Insulin Resistance; MACCE, major adverse cardiac and cerebrovascular events.

In the KNHANES, the differences between participants with low and normal‐low muscle masses were similar to those found in the NHANES. Korean adults with low muscle mass had significantly lower HOMA‐IR values (2.1 vs. 2.5, respectively; *p* < 0.001) than those with normal muscle mass. Participants with low muscle mass had a higher prevalence of malignancy (2.6% vs. 3.4%, respectively; *p* = 0.007), all‐cause mortality (5.5% vs. 12.1%, respectively; *p* < 0.001) and MACCE‐related mortality (1.3% vs. 2.8%, respectively; *p* < 0.001) than those with normal muscle mass.

### 3.2 Effects of Low Muscle Mass and IR on Mortality Risk

**FIGURE 1:**
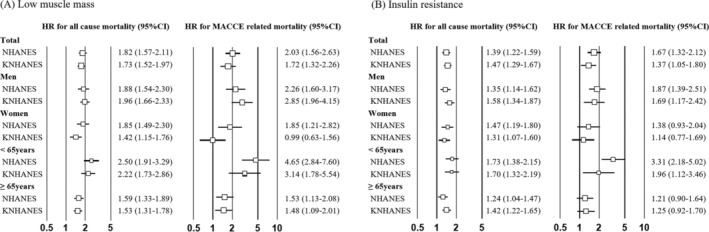
Cox regression analysis for mortality risks. (A) Low muscle mass; (B) insulin resistance. Adjusted with age, sex, race, smoking, alcohol consumption, history of cancer, hypertension, dyslipidaemia and eGFR.

The prevalence of IR was lower among participants with low muscle mass than in those with normal muscle mass in both cohorts (10.6% vs. 27.8% in the NHANES and 17.8% vs. 29.9% in the KNHANES, respectively). Correlation analysis revealed significant positive relationships between muscle mass and IR (*r* = 0.203, *p* < 0.001 in the NHANES; *r* = 0.143, *p* < 0.001 in the KNHANES). Increased muscle mass also showed an increased risk of IR in both adult cohorts (odds ratio [OR]: 1.97, 95%confidence interval [CI]: 1.87–1.66, *p* < 0.001 in the NHANES; OR: 1.75, 95% CI: 1.66–1.83, *p* < 0.001 in the KNHANES). The association between low muscle mass, IR and all‐cause and MACCE‐related mortality was assessed using Cox regression models. In the multivariate Cox regression model, low muscle mass and IR were independent risk factors for all‐cause and MACCE‐related mortality (Figure 1A).

In the subgroup analysis, low muscle mass was significantly associated with an increased risk of MACCE‐related mortality in adults younger than 65 years in both cohorts (HR: 4.65, 95% CI: 2.84–7.96 in the NHANES; HR: 3.14, 95% CI: 1.78–5.54 in the KNHANES). However, IR did not increase the risk of MACCE‐related mortality in women (HR: 1.38, 95% CI: 0.93–2.04 in the NHANES; HR: 1.14, 95% CI: 0.77–1.69 in the KNHANES). IR also did not increase the risk of MACCE‐related mortality in the older group (≥ 65 years) (HR: 1.21, 95% CI: 1.66–1.83 in the NHANES; HR: 1.23, 95% CI: 0.92–1.70 in the KNHANES; Figure 1B).

### 3.3 Effects of Low Muscle Mass on Mortality Risk According to IR

To comprehensively assess the impact of low muscle mass on mortality, we categorized study participants into four groups based on their muscle mass and IR status: normal muscle mass without IR (NM‐IS), low muscle mass without IR (LM‐IS), normal muscle mass with IR (NM‐IR) and low muscle mass with IR (LM‐IR) (Table 2). A multivariate Cox regression analysis was performed to examine the relationship between low muscle mass and mortality risk in the four groups (Table 2). Compared with the NM‐IS group as a reference, all other groups showed an increased risk of all‐cause mortality in both the US and Korean cohorts. Among them, the LM‐IR group had the highest risk of all‐cause mortality (HR: 2.30, 95% CI: 1.72–3.08 in the NHANES; HR: 2.60, 95% CI: 2.14–3.16 in the KNHANES). In addition, the LM‐IR group had the highest risk of MACCE‐related mortality among the groups (HR: 3.18, 95% CI: 1.99–5.08 in the NHANES; HR: 2.47, 95% CI: 1.66–3.69 in the KNHANES). The LM‐IR group also had the highest risk of all‐cause mortality in subgroup analyses stratified by age and race (Table S1 and Table S2).

**TABLE 2:** Effects of low muscle mass on mortality risk according to muscle mass and insulin resistance.

	All‐cause mortality		MACCE‐related mortality	
	HR (95% CI)	*p*	HR (95% CI)	*p*
NHANES				
NM‐IS	Reference			
LM‐IS	1.897 (1.608–2.238)	< 0.001	2.103 (1.551–2.851)	< 0.001
NM‐IR	1.436 (1.239–1.664)	< 0.001	1.722 (1.321–2.245)	< 0.001
LM‐IR	2.301 (1.719–3.078)	< 0.001	3.182 (1.994–5.079)	< 0.001
KNHANES				
NM‐IS	Reference			
LM‐IS	1.649 (1.401–1.941)	< 0.001	1.582 (1.122–2.232)	0.009
NM‐IR	1.397 (1.186–1.647)	< 0.001	1.261 (0.897–1.774)	0.182
LM‐IR	2.597 (2.138–3.156)	< 0.001	2.473 (1.659–3.687)	< 0.001

Abbreviations: LM‐IR, low muscle mass with insulin resistance; LM‐IS, low muscle mass without insulin resistance; NM‐IR, normal muscle mass with insulin resistance; NM‐IS, normal muscle mass without insulin resistance. Adjusted with age, sex, race, smoking, alcohol consumption, history of cancer, hypertension, dyslipidaemia and eGFR.

**FIGURE 2:**
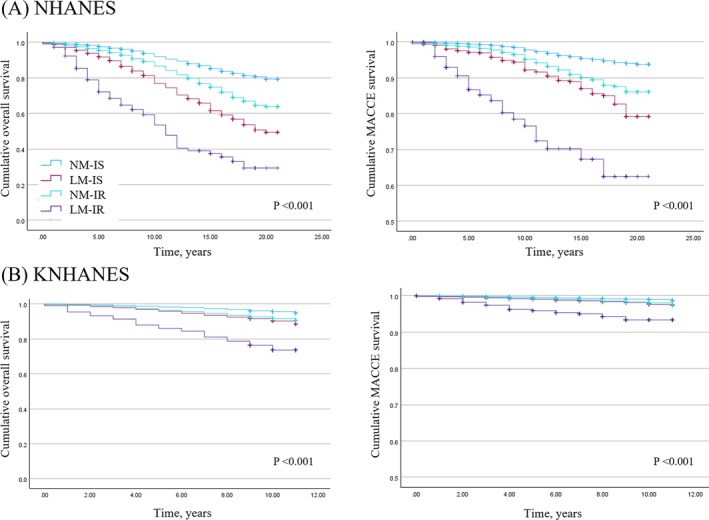
Kaplan–Meier survival curves of survival data in relation to low muscle mass. (A) NHANES; (B) KNHANES. LM‐IR, low muscle mass with insulin resistance; LM‐IS, low muscle mass without insulin resistance; NM‐IR, normal muscle mass with insulin resistance; NM‐IS, normal muscle mass without insulin resistance.

Survival probabilities among the four groups were plotted using the Kaplan–Meier method. Log‐rank tests were then performed, which revealed a statistically significant difference in survival distributions (log‐rank test, *p* < 0.001). Kaplan–Meier analysis revealed that individuals with low muscle mass and IR had a substantially higher risk of all‐cause and MACCE‐related mortality than those in other groups in both the US and Korean cohorts (Figure 2). In sex‐specific subgroup analyses, this increased risk remained consistent in both male and female populations. In particular, individuals with low muscle mass and IR had a significantly increased risk of all‐cause and MACCE‐related mortality in both cohorts, regardless of sex (Figures S2 and S3).

### 3.4 Mediation Analysis for the Effect of Low Muscle Mass on Mortality

**FIGURE 3:**
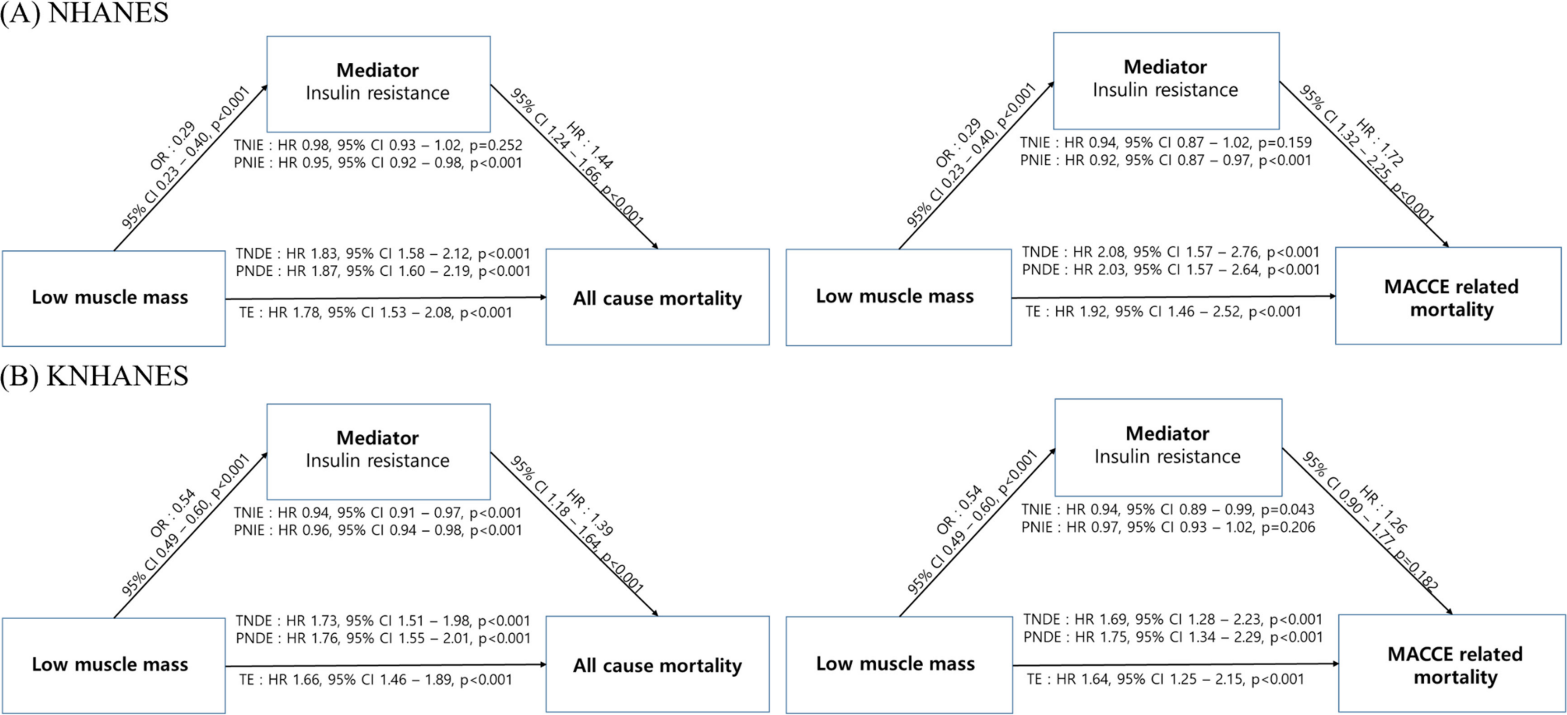
Mediation analysis of the effect of low muscle mass through insulin resistance on mortality. (A) NHANES; (B) KNHANES. TE, total effect; TNDE, total natural direct effect; TNIE, total natural indirect effect. Adjusted with age, sex, race, smoking, alcohol consumption, history of cancer, hypertension, dyslipidaemia and eGFR.

To further elucidate the relationships among low muscle mass, IR and mortality, a mediation analysis was performed (Figure 3). In the NHANES, the total effect of low muscle mass on all‐cause mortality was significant (HR: 1.78, 95% CI: 1.53–2.07). Low muscle mass was associated with reduced IR (HR: 0.29, 95% CI: 0.23–0.40) and a consequent reduction in the risk of all‐cause mortality (PNIE:HR: 0.95, 95% CI: 0.92–0.97). However, low muscle mass directly increased the risk of all‐cause mortality (TNDE: HR: 1.87, 95% CI: 1.60–2.19). For MACCE‐related mortality, low muscle mass was associated with a reduced risk via insulin resistance (PNIE: HR: 0.92, 95% CI: 0.87–0.97) but had a direct association with an increased risk of MACCE‐related mortality (TNDE: HR: 2.08, 95% CI: 1.57–2.76) (Figure 3A).

In the KNHANES, the total effect of low muscle mass on all‐cause mortality was statistically significant (HR: 1.66, 95% CI: 1.45–1.89). Specifically, low muscle mass was associated with reduced IR (HR: 0.54, 95% CI: 0.49–0.60), which in turn was associated with a reduced risk of all‐cause mortality (PNIE:HR: 0.96, 95% CI: 0.94–0.98). Despite this indirect protective effect, low muscle mass was directly associated with an increased risk of all‐cause mortality (TNDE: HR: 1.73, 95% CI: 1.52–1.98). For MACCE‐related mortality, low muscle mass was similarly associated with a reduced risk through IR (PNIE:HR: 0.97, 95% CI: 0.93–1.01) but was directly associated with a higher risk of MACCE‐related mortality (TNDE: HR: 1.69, 95% CI: 1.28–2.23; Figure 3B).

### 3.5 Effects of Low Relative Muscle Mass on IR and Mortality Risk

Body weight has been shown to be significantly associated with skeletal muscle mass as well as IR [38]. Therefore, we assessed participants' relative muscle mass using the ASM/body mass and aMFR indices, which adjust for body weight and fat mass, respectively, to better understand their effects on IR and mortality risk. Low ASM/body mass showed a significantly increased risk of all‐cause mortality (HR: 1.379, 95% CI: 1.209–1.573) (Table 3). Mediation analysis was then performed (Figure S4). The overall effect of low relative muscle mass (ASM/body mass) on all‐cause mortality was statistically significant (HR: 1.37, 95% CI: 1.20–1.56). Low ASM/body mass was associated with increased IR (HR: 2.72, 95% CI: 2.40–3.07), and increased IR mediated an increased risk of all‐cause mortality (PNIE: HR: 1.04, 95% CI: 1.00–1.07). Low ASM/body mass was directly associated with an increased risk of all‐cause mortality (TNDE: HR: 1.32, 95% CI: 1.16–1.51) (Figure S4).

**TABLE 3:** Effects of low relative muscle mass (ASM/body weight) on mortality risk in the NHANES cohort.

	ASM/body weight		aMFR	
	HR (95% CI)	*p*	HR (95% CI)	*p*
Total	1.379 (1.209–1.573)	< 0.001	1.148 (1.010–1.306)	0.035
Male	1.439 (1.197–1.730)	< 0.001	1.161 (0.974–1.384)	0.096
Female	1.390 (1.147–1.685)	0.001	1.190 (0.982–1.441)	0.076
Premenopausal status	1.731 (0.682–4.393)	0.248	1.349 (0.514–3.539)	0.543
Menopausal status	1.397 (1.138–1.714)	0.001	1.244 (1.015–1.525)	0.036
≥ 65 years	1.182 (1.004–1.393)	0.045	1.024 (0.874–1.200)	0.77
< 65 years	1.852 (1.488–2.306)	0.001	1.438 (1.156–1.790)	0.001
Non‐Hispanic White	1.275 (1.082–1.503)	0.004	1.072 (0.912–1.259)	0.4
Non‐Hispanic Black	1.209 (0.796–1.837)	0.374	0.959 (0.648–1.420)	0.835
Hispanic	1.685 (1.254–2.264)	0.059	2.323 (0.961–5.614)	0.061

Abbreviations: aMFR, appendicular muscle mass divided by total body fat mass; ASM, appendicular skeletal muscle mass. Adjusted with age, sex, race, smoking, alcohol consumption, history of cancer, hypertension, dyslipidaemia and eGFR.

Similar to ASM/body mass, low aMFR showed a significant association with all‐cause mortality (HR: 1.148, 95% CI: 1.01–1.306) (Table 3). However, it was not a significant risk factor in the older group (≥ 65 years, HR: 1.024, 95% CI: 0.874–1.2) in the subgroup analysis (Table 3). In the mediation analysis, the effect of low aMFR on all‐cause mortality was statistically significant (HR: 1.16, 95% CI: 1.02–1.32). Low aMFR was also associated with increased IR (HR: 2.72, 95% CI: 2.42–3.07), and increased IR mediated an increased risk of all‐cause mortality (PNIE: HR: 1.04, 95% CI: 1.01–1.08). However, low aMFR did not show a direct association with an increased risk of all‐cause mortality (TNDE: HR: 1.11, 95% CI: 0.97–1.26) (Figure S4). Taken together, these findings suggest that low muscle mass relative to body weight or fat increases mortality risk by worsening insulin resistance, highlighting the complex interaction between muscle and fat in regulating metabolic health.

## 4 Discussion

In this study, we thoroughly examined the associations among muscle mass and IR and their combined effects on all‐cause and MACCE‐related mortality in adult cohorts from the United States and Korea. To the best of our knowledge, this is the first multi‐nationwide, population‐based study to compare the all‐cause and MACCE‐related mortality risk across cohorts stratified by baseline skeletal muscle mass, metabolic health and IR. Our study uniquely used mediation analysis to elucidate the direct and indirect effects of low muscle mass on mortality in two distinct populations, thereby providing novel insights into the pathophysiological mechanisms underlying these associations.

Consistent with previous research, our results show that low muscle mass is an independent risk factor for all‐cause and MACCE‐related mortality [39, 40] [S1]. The group with both low muscle mass and IR had the highest risk of all‐cause and MACCE‐related mortality in both the US and Korean cohorts, highlighting the compounded risk of these conditions. Furthermore, in both cohorts, even in adults aged less than 65 years, adults with low muscle mass had a 4.6 and 3.1 times higher risk of MACCE‐related mortality than those with normal muscle mass in the US and Korean cohorts, respectively. This highlights the importance of early detection and intervention to maintain muscle mass, especially in patients aged less than 65 years.

Kaplan–Meier survival analysis showed that the LM‐IR group had the poorest prognosis in both the US and Korean cohorts. Notably, the LM‐IR group in the US cohort had a lower survival probability than the LM‐IR group in the Korean cohort (Figure 2). This discrepancy might be a reflection of the differences in the prevalence of hypertension between the two cohorts (Table 1). In the US cohort, the low muscle mass group had a significantly higher prevalence of hypertension (40.7%) compared with the Korean cohort (19.8%). Hypertension is a well‐established risk factor for both cardiovascular and all‐cause mortality [S2], and its higher prevalence in the US cohort likely contributed to the reduced survival probabilities observed in this group. Recent epidemiological studies also support this observation, showing that the prevalence of hypertension in the United States was 49.6% in 2017–2018 [S3]. In contrast, the prevalence of hypertension in Korean adults is significantly lower at approximately 28% [S4].

Previous studies have indicated a pathogenic role of sarcopenia in the development of IR. Factors such as myocyte lipid accumulation, mitochondrial dysfunction, inflammation and a decreased proportion of Type I muscle fibres in sarcopenic muscles contribute to increased IR [12, 16]. However, our study found that muscle mass was positively associated with IR, increasing the risk of IR by 1.9 and 1.7 times in the US and Korean cohorts, respectively. This association remained significant in multivariate analyses including body weight as a variable. Bijlsma et al. reported similar results showing that absolute muscle mass was positively associated with insulin secretion and HOMA‐IR in a Netherlands cohort study [S5]. In this Netherlands cohort, absolute muscle mass was positively associated with insulin secretion and HOMA‐IR. This counterintuitive finding may be because higher muscle mass is often associated with higher body and fat mass, both of which are known to contribute to IR. A recent cohort study in Hong Kong showed similar findings, with a positive association between muscle mass, body mass index and body weight. In this study, obesity was identified as a protective factor against sarcopenia, further complicating the association between muscle mass and metabolic health [25].

Similar results were found in a recent Korean adult cohort study [S6]. In this cohort study of Korean adults, participants were divided into four groups based on muscle and fat composition: low muscle/low fat, low muscle/high fat, high muscle/low fat and high muscle/high fat. The high‐muscle/high‐fat group had a significantly higher incidence of metabolic syndrome than the low‐muscle/high‐fat group (36% vs. 19.9%). This finding may be attributed to myosteatosis, which involves the accumulation of intramyocellular lipids and has been associated with impaired insulin sensitivity [S7, S8]. Animal studies have provided mechanistic insight into this finding. Feeding a high‐fat diet (HFD) resulted in increased intramyocellular lipid accumulation in both young and old mice [S9]. Furthermore, HFD‐fed mice showed a significant decline in muscle function, including reduced grip strength and impaired sensorimotor coordination [S10]. These findings highlight the need to assess both muscle mass and quality when evaluating sarcopenic individuals.

A recent notable imaging study from the UK Biobank examined the effect of muscle fat infiltration on all‐cause mortality through the assessment of muscle composition using magnetic resonance imaging (MRI) [S11]. This study found that participants with high levels of muscle fat infiltration, even in the presence of normal muscle volume, had a significantly higher risk of all‐cause mortality compared with those with normal fat infiltration and normal muscle volume. In addition, participants with normal fat infiltration but low muscle volume had a lower risk of mortality compared with those with high muscle fat infiltration, highlighting the deleterious effects of myosteatosis. These findings underscore the critical role of muscle quality, particularly fat infiltration, in predicting survival outcomes in people with sarcopenia.

To unveil this complex association, mediation analysis provided a better understanding of the indirect pathways through which low muscle mass affects mortality. Although IR increased the risk of all‐cause mortality in both American and Korean adults, low muscle mass was associated with reduced IR, which was paradoxically associated with a lower risk of all‐cause mortality. Nevertheless, the direct effect of low muscle mass on the increased risk of death was still significant in both cohorts, suggesting that factors related to the loss of muscle mass contribute to mortality, in addition to IR [40]. Sarcopenia can lead to poor physical performance, malnutrition, decreased metabolic rate, increased frailty, higher fracture risk, increased adipose tissue inflammation and more frequent hospitalizations, all of which are critical determinants of health in older adults [40] [S12, S13].

Moreover, the different results between the US and Korean cohorts regarding the effect of IR on MACCE‐related mortality are also remarkable. In the US cohort, IR was a significant predictor of MACCE‐related mortality, whereas this association was not observed in the Korean cohort. However, a different Korean cohort study (the Korean Genome and Epidemiology Study) reported a higher cumulative incidence of CVD in participants with IR than in those without [S14]. This discrepancy may reflect differences in the genetic, environmental and lifestyle factors that influence metabolic health in these populations, highlighting the complexity of metabolic health and its interaction with CVD outcomes.

There are currently no FDA‐approved treatments for sarcopenia [S15]. Although several pharmacologic agents have been studied, including landogrozumab and bimagrumab, which target myostatin, they have shown improvements in lean body mass but have failed to significantly improve muscle function in clinical trials [S16]. Other agents such as testosterone, growth hormone, selective androgen receptor modulators (SARMs), dehydroepiandrosterone (DHEA) and vitamin D have also shown limited efficacy in improving muscle function in clinical trials [S17]. However, emerging research on exercise‐induced circulating factors (exerkines) such as irisin and apelin provide promising therapeutic opportunities. These molecules have demonstrated the potential to improve muscle function and ameliorate metabolic dysfunction in preclinical models of sarcopenia [S18, S19]. Bimagrumab is currently being evaluated in combination with semaglutide, a GLP‐1 receptor agonist, to explore its potential in treating obesity while preserving muscle mass. Future therapeutic strategies targeting insulin resistance and metabolic dysfunction could potentially have an impact on sarcopenia and mortality, in view of the interplay between these factors observed in the present study.

This study has several strengths, including the use of large multinational cohorts (NHANES and KNHANES), which enhance the generalizability of the findings across diverse populations. This comprehensive analysis goes beyond simple correlations by utilizing a mediation analysis to elucidate the direct and indirect effects of low muscle mass and IR on mortality. Clinically, this highlights the independent and synergistic effects of sarcopenia and IR on all‐cause and MACCE‐related mortality, underscoring the need for targeted intervention strategies. Additionally, a detailed examination of population characteristics, including age and sex, emphasized the importance of early detection and intervention across ethnic and regional backgrounds.

This study has a few limitations. First, the cross‐sectional nature of the NHANES and KNHANES data limited the ability to establish causality between muscle mass, IR and mortality outcomes. Therefore, although we found that sarcopenia was positively associated with cancer in both cohorts, we did not perform further analyses because of the difficulty in assessing casualties. Longitudinal studies are required to confirm these associations. Second, the definitions and measurement techniques for muscle mass and IR may differ between populations, potentially affecting the comparability of the results. Third, residual confounding factors such as dietary intake, physical activity levels and other comorbidities may have influenced the observed associations. In addition, our study showed a positive association between muscle mass and IR. We hypothesize that this finding may be due to myosteatosis, which may impair insulin sensitivity despite higher muscle mass. However, this hypothesis requires further validation. Future studies should include detailed muscle quality assessments, such as MRI [S20], along with detailed measurements of insulin resistance using both fasting and postprandial glucose levels, such as the Matsuda insulin sensitivity index [S21]. These methods will provide a deeper understanding of how fat infiltration affects muscle function and insulin sensitivity.

In conclusion, our study provides valuable insights into the intricate relationships among muscle mass (ASMI), IR and mortality risk and highlights the need for targeted interventions to improve health outcomes in the aging population, particularly in younger adults with sarcopenia and IR. Future studies should aim to elucidate the underlying mechanisms and assess the efficacy of combined interventions in reducing the global health burden of sarcopenia and metabolic diseases. Addressing these critical health issues may improve the quality of life and reduce mortality among older adults, ultimately contributing to healthier aging populations worldwide.

## 5 Limitation

Our study found a positive association between muscle mass and insulin resistance, which is inconsistent with previous research suggesting that sarcopenia contributes to insulin resistance. This difference could be due to differences in population characteristics, body composition or unmeasured factors such as myosteatosis. Further studies, including MRI imaging and mechanistic research, are needed to better understand this relationship.

## Ethics Statement

The study protocol was approved by the Institutional Review Board (IRB) of Gwangju Institute of Science and Technology (#20221201‐BR‐69‐02‐02) and Seoul National University Bundang Hospital (IRB number: X‐2307‐840‐902). Ethical approval for the studies was provided by two separate review boards: the Research Ethics Review Board of the National Center for Health Statistics and the US Centers for Disease Control and Prevention, which approved all NHANES protocols (NCHS IRB/ERB Protocol Number: 1999–2004, Protocol #98–12; 2005–2010, Protocol #2005–06; 2011–2016, Protocol #2011–17), and the IRB of the KCDC, which granted approval for all KNHANES protocols (2008‐04EXP‐01‐C, 2009‐01CON‐03‐2C, 2010‐02CON21‐C, KCDC‐2011‐02CON‐06‐C). All participants volunteered and provided written informed consent before enrolment. Patient records were anonymized before they were accessed by the authors. All the analyses were performed in accordance with the principles of the Declaration of Helsinki.

## Conflicts of Interest

The authors declare no conflicts of interest.

## Supporting information


**Figure S1** Flowchart for final selection.
Figure S2 Kaplan–Meier survival curves of overall survival data in relation to low muscle mass in male and female. Abbreviations: NM‐IS, normal muscle mass without insulin resistance; LM‐IS, low muscle mass without insulin resistance; NM‐IR, normal muscle mass with insulin resistance; LM‐IR, low muscle mass with insulin resistance.
Figure S3 Kaplan–Meier survival curves of MACCE survival data in relation to low muscle mass in male and female. Abbreviations: MACCE, major adverse cardiovascular and cerebrovascular events; NM‐IS, normal muscle mass without insulin resistance; LM‐IS, low muscle mass without insulin resistance; NM‐IR, normal muscle mass with insulin resistance; LM‐IR, low muscle mass with insulin resistance.
Figure S4 Mediation analysis of the effect of low relative muscle mass through insulin resistance on mortality. Abbreviations: ASM, appendicular skeletal muscle mass; aMFR, appendicular muscle mass divided by total body fat mass; TNIE, total natural indirect effect; TNDE, total natural direct effect; TE, total effect. Adjusted with age, sex, race, smoking, alcohol consumption, history of cancer, hypertension, dyslipidaemia and eGFR.
Table S1 Effects of low muscle mass on mortality risk according to muscle mass and insulin resistance in people ≥ 65 years. Abbreviations: NM‐IS, normal muscle mass without insulin resistance; LM‐IS, low muscle mass without insulin resistance; NM‐IR, normal muscle mass with insulin resistance; LM‐IR, low muscle mass with insulin resistance. Adjusted with age, sex, race, smoking, alcohol consumption, history of cancer, hypertension, dyslipidaemia and eGFR.
Table S2 Effects of low muscle mass on mortality risk by muscle mass and insulin resistance by Race. Abbreviations: NM‐IS, normal muscle mass without insulin resistance; LM‐IS, low muscle mass without insulin resistance; NM‐IR, normal muscle mass with insulin resistance; LM‐IR, low muscle mass with insulin resistance. Adjusted with age, sex, race, smoking, alcohol consumption, history of cancer, hypertension, dyslipidaemia and eGFR.

## Data Availability

The data for this study are available from the corresponding author upon reasonable request.
