# Supplementary material for: Association of Appendicular Skeletal Muscle Mass Index and Insulin Resistance With Mortality in Multi‐Nationwide Cohorts
Source: J Cachexia Sarcopenia Muscle. 2025 Apr 14;16(2):e13811. doi: 10.1002/jcsm.13811 (PMC11997253; doi:10.1002/jcsm.13811)
Supplement: Supplementary file 1 — Figure S1 Flowchart for final selection. Figure S2 Kaplan–Meier survival curves of overall survival data in relation to low muscle mass in male and female. Abbreviations: NM‐IS, normal muscle mass without insulin resistance; LM‐IS, low muscle mass without insulin resistance; NM‐IR, normal muscle mass with insulin resistance; LM‐IR, low muscle mass with insulin resistance. Figure S3 Kaplan–Meier survival curves of MACCE survival data in relation to low muscle mass in male and female. Abbreviations: MACCE, major adverse cardiovascular and cerebrovascular events; NM‐IS, normal muscle mass without insulin resistance; LM‐IS, low muscle mass without insulin resistance; NM‐IR, normal muscle mass with insulin resistance; LM‐IR, low muscle mass with insulin resistance. Figure S4 Mediation analysis of the effect of low relative muscle mass through insulin resistance on mortality. Abbreviations: ASM, appendicular skeletal muscle mass; aMFR, appendicular muscle mass divided by total body fat mass; TNIE, total natural indirect effect; TNDE, total natural direct effect; TE, total effect. Adjusted with age, sex, race, smoking, alcohol consumption, history of cancer, hypertension, dyslipidaemia and eGFR. Table S1 Effects of low muscle mass on mortality risk according to muscle mass and insulin resistance in people ≥ 65 years. Abbreviations: NM‐IS, normal muscle mass without insulin resistance; LM‐IS, low muscle mass without insulin resistance; NM‐IR, normal muscle mass with insulin resistance; LM‐IR, low muscle mass with insulin resistance. Adjusted with age, sex, race, smoking, alcohol consumption, history of cancer, hypertension, dyslipidaemia and eGFR. Table S2 Effects of low muscle mass on mortality risk by muscle mass and insulin resistance by Race. Abbreviations: NM‐IS, normal muscle mass without insulin resistance; LM‐IS, low muscle mass without insulin resistance; NM‐IR, normal muscle mass with insulin resistance; LM‐IR, low muscle mass with insulin resistance. Adjusted wit [file JCSM-16-e13811-s001.docx]

Supplementary Figure 1. Flowchart for final selection


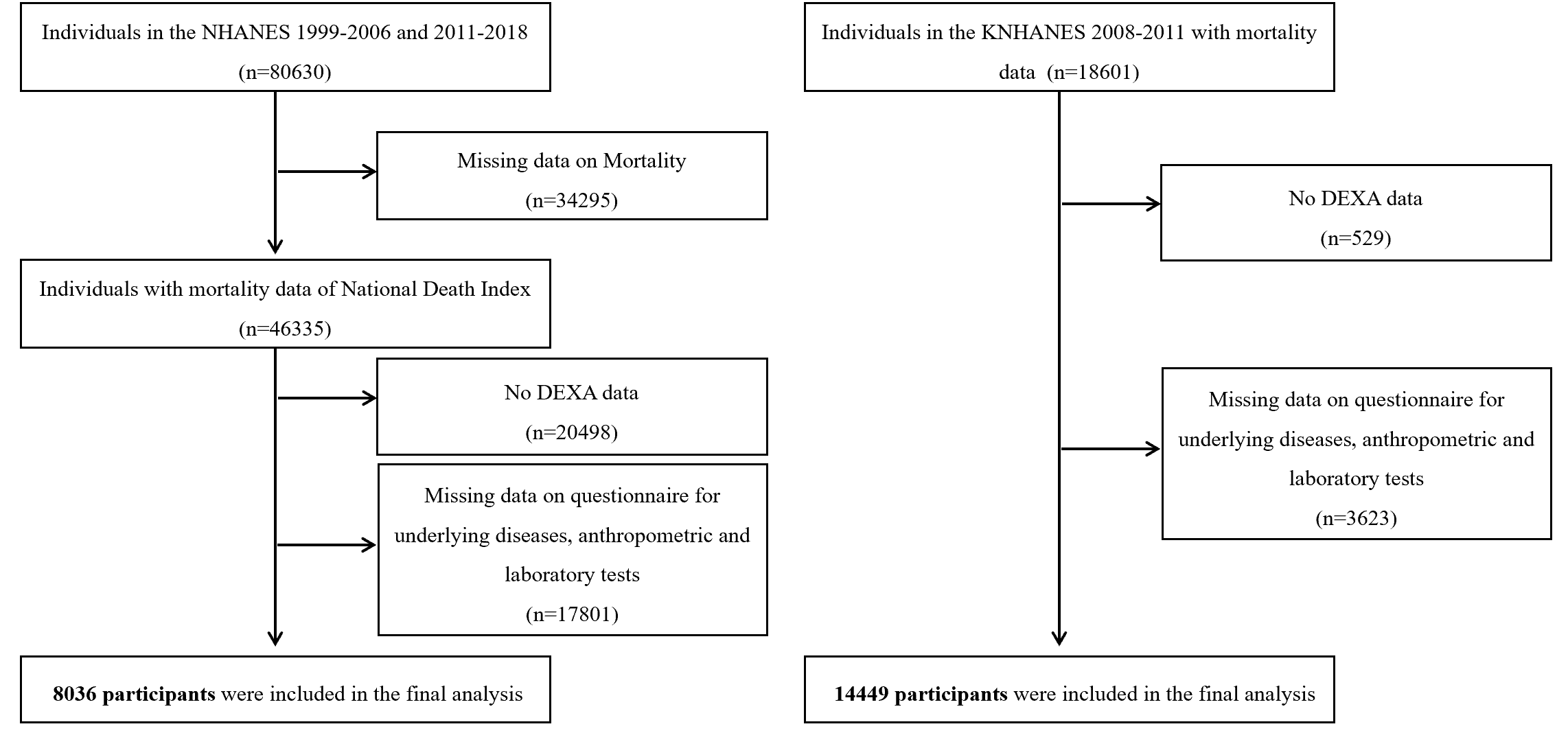


Supplementary Figure 2. Kaplan-Meier survival curves of overall survival data in relation to low muscle mass in male and female


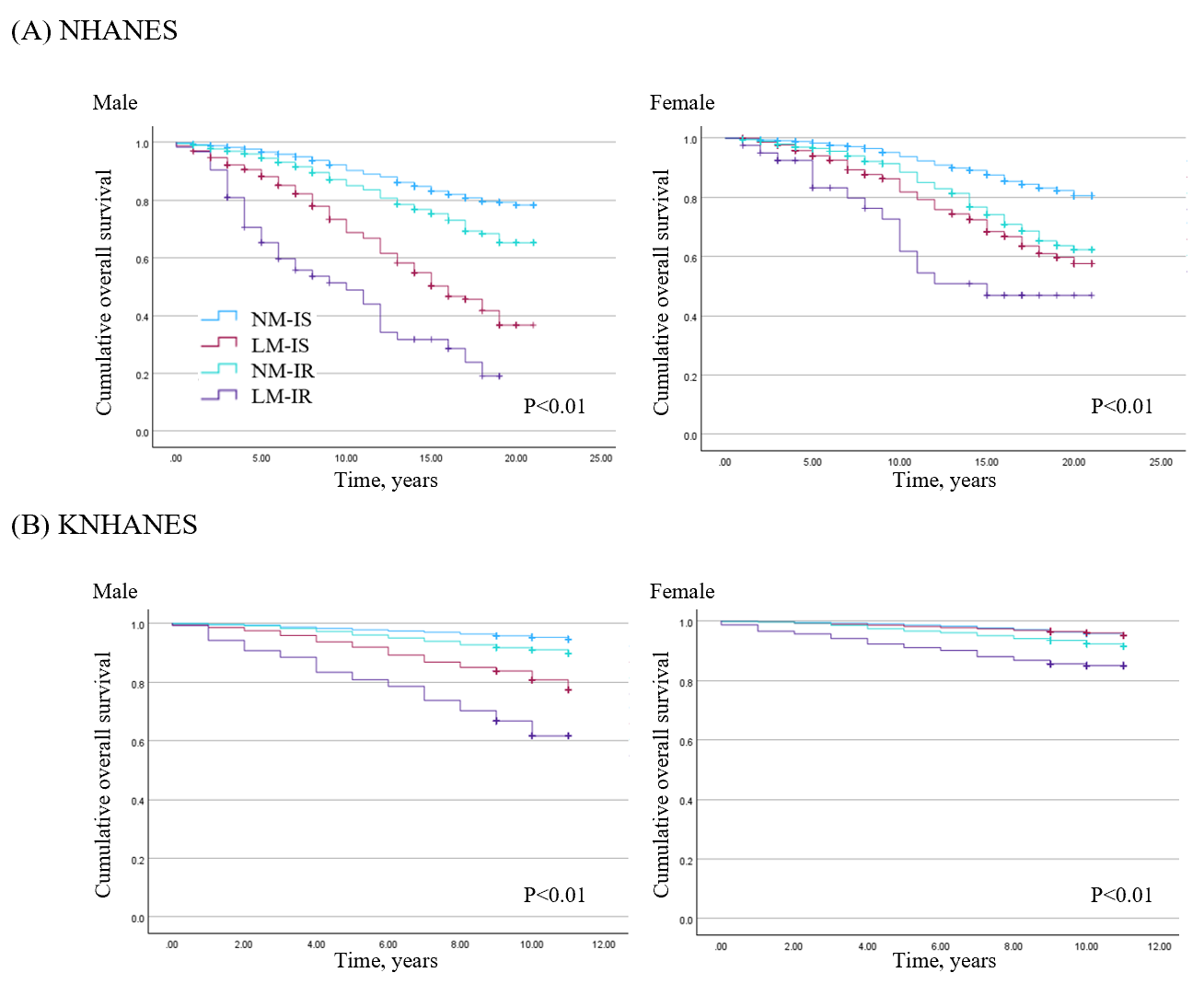


Abbreviations: NM-IS, normal muscle mass without insulin resistance; LM-IS, low muscle mass without insulin resistance; NM-IR, normal muscle mass with insulin resistance; LM-IR, low muscle mass with insulin resistance

Supplementary Figure 3. Kaplan-Meier survival curves of MACCE survival data in relation to low muscle mass in male and female


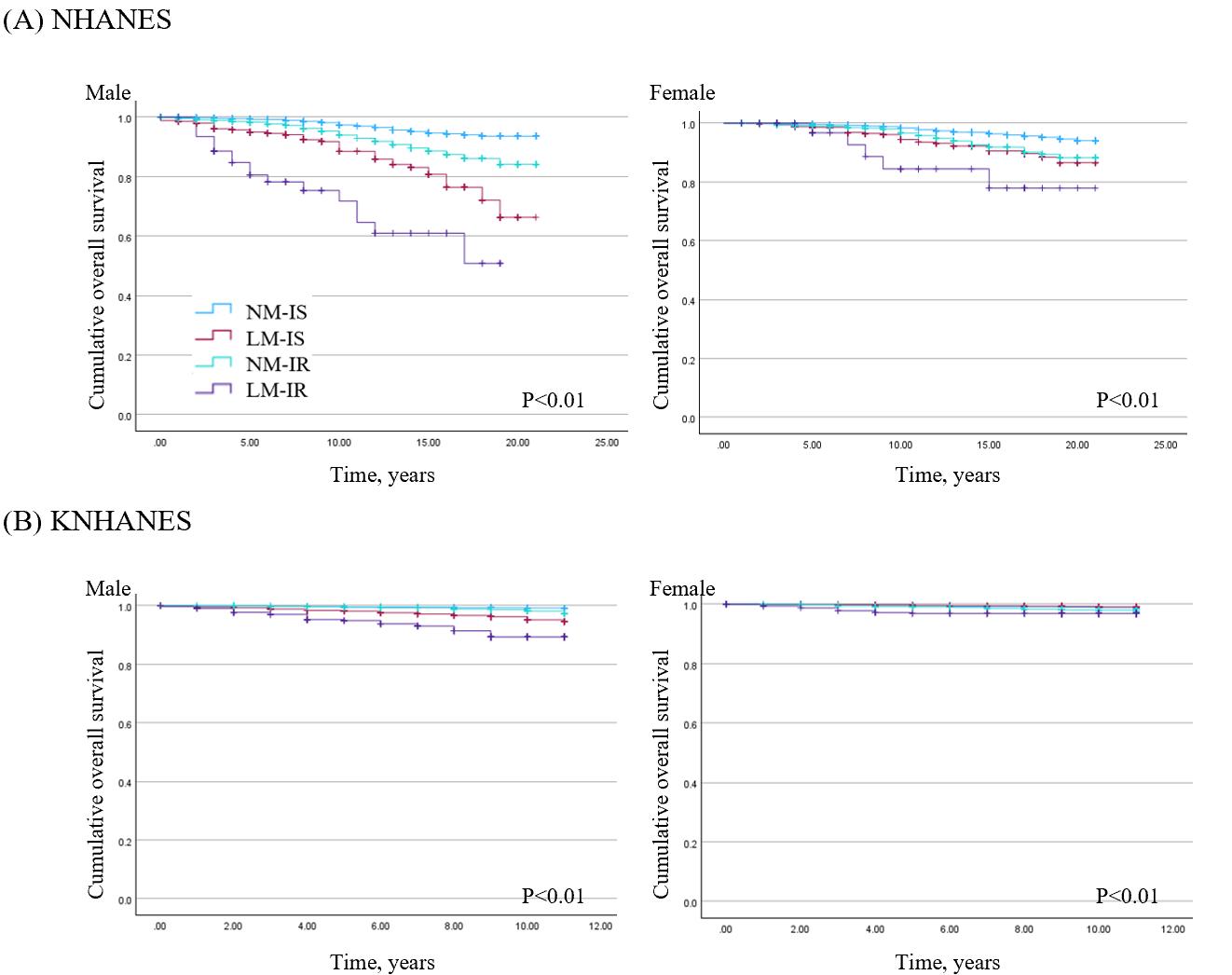


Abbreviations: MACCE, major adverse cardiovascular and cerebrovascular events; NM-IS, normal muscle mass without insulin resistance; LM-IS, low muscle mass without insulin resistance; NM-IR, normal muscle mass with insulin resistance; LM-IR, low muscle mass with insulin resistance

Supplementary Figure 4. Mediation Analysis of effect of low relative muscle mass through insulin resistance on mortality


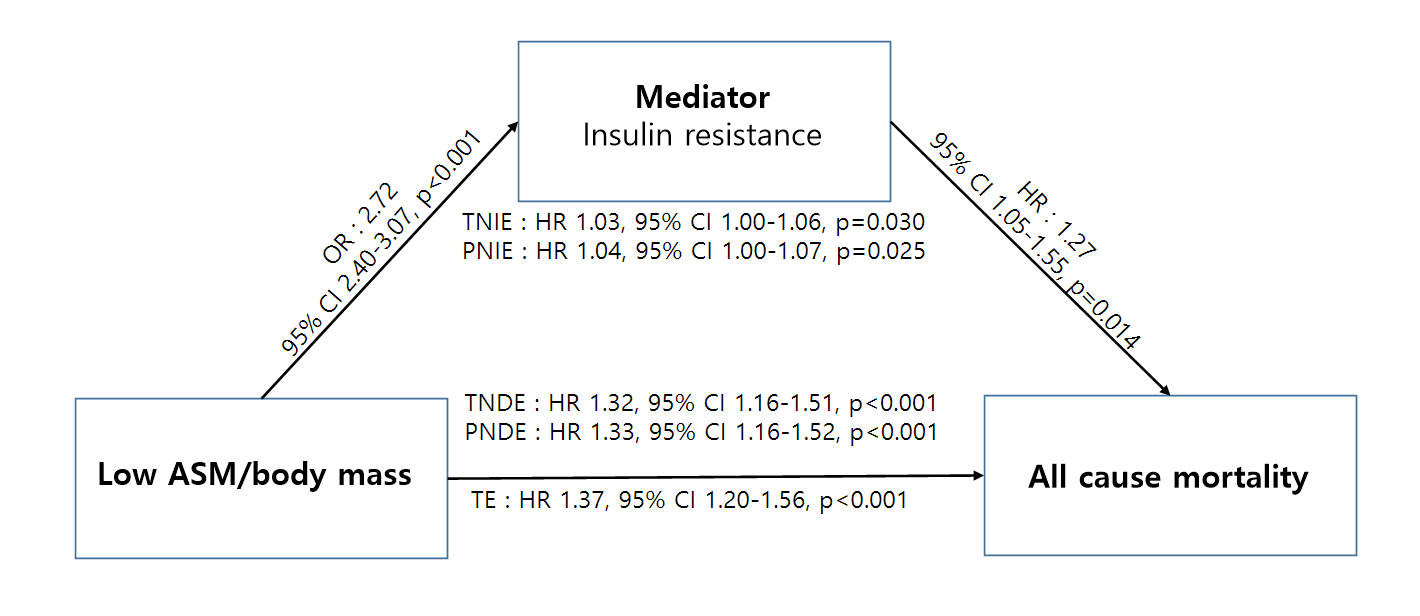


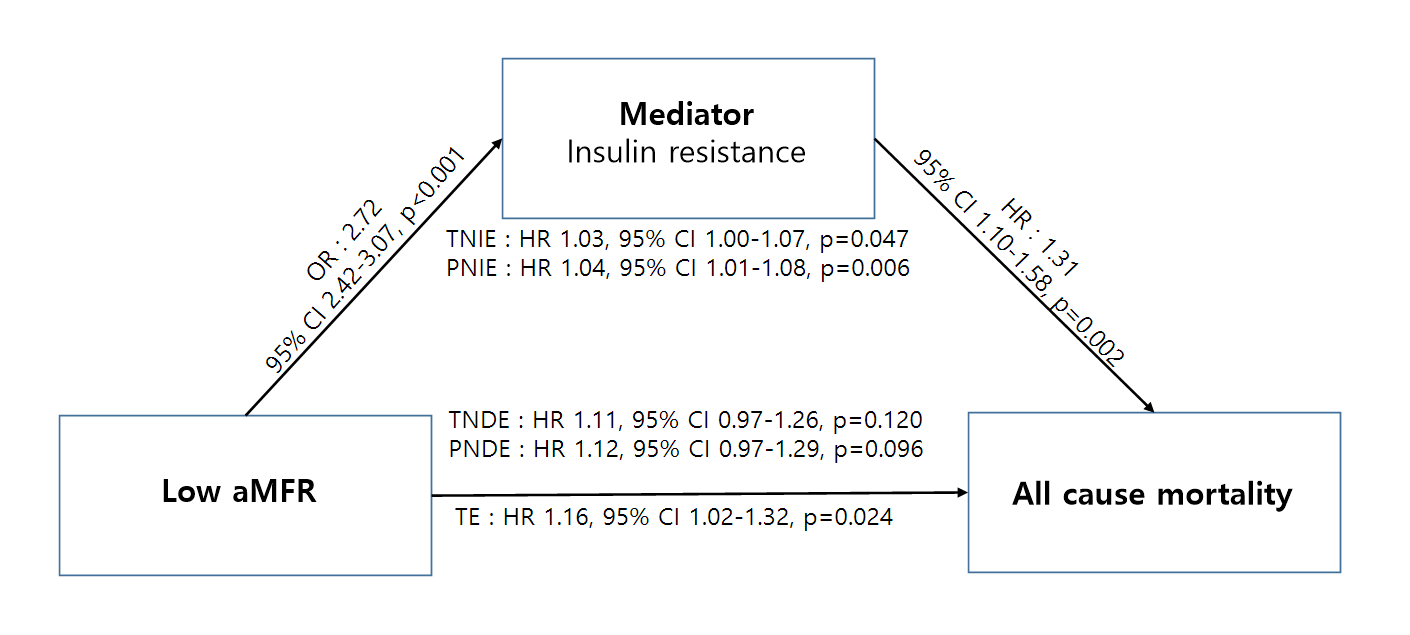


Abbreviations: ASM, appendicular skeletal muscle mass; aMFR, appendicular muscle mass divided by total body fat mass; TNIE, total natural indirect effect; TNDE, total natural direct effect; TE, total effect. Adjusted with age, sex, race, smoking, alcohol consumption, history of cancer, hypertension, dyslipidemia, and eGFR

Supplementary Table 1. Effects of low muscle mass on mortality risk according to muscle mass and insulin resistance in people ≥ 65 years

|  | NHANES | |  | KNHANES | |
| --- | --- | --- | --- | --- | --- |
|  | HR (95% CI) | *P* |  | HR (95% CI) | *P* |
| ≥ 65 years |  |  |  |  |  |
| NM-IS | Reference |  |  | Reference |  |
| LM-IS | 1.641 (1.347-1.999) | <0.001 |  | 1.307 (1.075-1.589) | 0.007 |
| NM-IR | 1.289 (1.064-1.562) | 0.01 |  | 1.218 (0.999-1.484) | 0.051 |
| LM-IR | 1.729 (1.228-2.433) | 0.002 |  | 2.263 (1.822-2.810) | <0.001 |
| < 65 years |  |  |  |  |  |
| NM-IS | Reference |  |  | Reference |  |
| LM-IS | 2.331 (1.718-3.162) | <0.001 |  | 2.533 (1.878-3.416) | <0.001 |
| NM-IR | 1.651 (1.307-2.085) | <0.001 |  | 1.927 (1.434-2.590) | <0.001 |
| LM-IR | 5.081 (2.918-8.850) | <0.001 |  | 3.143 (1.957-5.047) | <0.001 |

Abbreviations: NM-IS, normal muscle mass without insulin resistance; LM-IS, low muscle mass without insulin resistance; NM-IR, normal muscle mass with insulin resistance; LM-IR, low muscle mass with insulin resistance. Adjusted with age, sex, race, smoking, alcohol consumption, history of cancer, hypertension, dyslipidemia, and eGFR

Supplementary Table 2. Effects of low muscle mass on mortality risk by muscle mass and insulin resistance by Race

|  | NHANES | |
| --- | --- | --- |
|  | HR (95% CI) | *P* |
| Non-Hispanic white |  |  |
| NM-IS | Reference |  |
| LM-IS | 1.777 (1.462-2.161) | <0.001 |
| NM-IR | 1.377 (1.125-1.685) | 0.002 |
| LM-IR | 2.066 (1.388-3.076) | <0.001 |
| Non-Hispanic black |  |  |
| NM-IS | Reference |  |
| LM-IS | 2.781 (1.482-5.217) | <0.001 |
| NM-IR | 1.703 (1.225-2.368) | 0.002 |
| LM-IR | 4.273 (1.034-17.664) | 0.045 |
| Hispanic |  |  |
| NM-IS | Reference |  |
| LM-IS | 1.863 (1.240-2.799) | 0.003 |
| NM-IR | 1.208 (0.876-1.666) | 0.248 |
| LM-IR | 2.243 (1.374-3.661) | 0.001 |

Abbreviations: NM-IS, normal muscle mass without insulin resistance; LM-IS, low muscle mass without insulin resistance; NM-IR, normal muscle mass with insulin resistance; LM-IR, low muscle mass with insulin resistance. Adjusted with age, sex, race, smoking, alcohol consumption, history of cancer, hypertension, dyslipidemia, and eGFR

Supplement References

S1. Zhang X, Wang C, Dou Q, Zhang W, Yang Y, Xie X. Sarcopenia as a predictor of all-cause mortality among older nursing home residents: a systematic review and meta-analysis. BMJ Open. 2018;8:e021252.

S2. Aune D, Huang W, Nie J, Wang YJBRI. Hypertension and the risk of all‐cause and cause‐specific mortality: an outcome‐wide association study of 67 causes of death in The National Health Interview Survey. 2021;2021:9376134.

S3. Chobufo MD, Gayam V, Soluny J, Rahman EU, Enoru S, Foryoung JB, et al. Prevalence and control rates of hypertension in the USA: 2017–2018. 2020;6:100044.

S4. Kim HC, Lee H, Lee H-H, Son D, Cho M, Shin S, et al. Korea Hypertension Fact Sheet 2023: analysis of nationwide population-based data with a particular focus on hypertension in special populations. 2024;30:7.

S5. Bijlsma AY, Meskers CG, van Heemst D, Westendorp RG, de Craen AJ, Maier AB. Diagnostic criteria for sarcopenia relate differently to insulin resistance. Age (Dordr). 2013;35:2367-75.

S6. Kim K, Park SMJSr. Association of muscle mass and fat mass with insulin resistance and the prevalence of metabolic syndrome in Korean adults: a cross-sectional study. 2018;8:2703.

S7. Sinha R, Dufour S, Petersen KF, LeBon V, Enoksson S, Ma Y-Z, et al. Assessment of skeletal muscle triglyceride content by 1H nuclear magnetic resonance spectroscopy in lean and obese adolescents: relationships to insulin sensitivity, total body fat, and central adiposity. 2002;51:1022-7.

S8. Kim H-K, Kim C-HJE, Metabolism. Quality matters as much as quantity of skeletal muscle: clinical implications of myosteatosis in cardiometabolic health. 2021;36:1161-74.

S9. Messa GA, Piasecki M, Hurst J, Hill C, Tallis J, Degens HJJoEB. The impact of a high-fat diet in mice is dependent on duration and age, and differs between muscles. 2020;223:jeb217117.

S10. Lee S-R, Khamoui AV, Jo E, Park B-S, Zourdos MC, Panton LB, et al. Effects of chronic high-fat feeding on skeletal muscle mass and function in middle-aged mice. 2015;27:403-11.

S11. Linge J, Petersson M, Forsgren MF, Sanyal AJ, Dahlqvist Leinhard OJJoc, sarcopenia, muscle. Adverse muscle composition predicts all‐cause mortality in the UK Biobank imaging study. 2021;12:1513-26.

S12. Chen H, Huang X, Dong M, Wen S, Zhou L, Yuan X. The Association Between Sarcopenia and Diabetes: From Pathophysiology Mechanism to Therapeutic Strategy. Diabetes Metab Syndr Obes. 2023;16:1541-54.

S13. Zhang X, Xie X, Dou Q, Liu C, Zhang W, Yang Y, et al. Association of sarcopenic obesity with the risk of all-cause mortality among adults over a broad range of different settings: a updated meta-analysis. BMC Geriatr. 2019;19:183.

S14. Lee JH, Lee HS, Jeon S, Lee YJ, Park B, Lee JH, et al. Association between experience of insulin resistance and long-term cardiovascular disease risk: findings from the Korean Genome and Epidemiology Study (KOGES). Endocrine. 2024;84:481-9.

S15. Evans WJ, Guralnik J, Cawthon P, Appleby J, Landi F, Clarke L, et al. Sarcopenia: no consensus, no diagnostic criteria, and no approved indication—How did we get here? 2024;46:183-90.

S16. Tsai S-YJTiMM. Lost in translation: challenges of current pharmacotherapy for sarcopenia. 2024;

S17. Rolland Y, Dray C, Vellas B, Barreto PDSJM. Current and investigational medications for the treatment of sarcopenia. 2023;149:155597.

S18. Vinel C, Lukjanenko L, Batut A, Deleruyelle S, Pradere J-P, Le Gonidec S, et al. The exerkine apelin reverses age-associated sarcopenia. 2018;24:1360-71.

S19. Guo M, Yao J, Li J, Zhang J, Wang D, Zuo H, et al. Irisin ameliorates age‐associated sarcopenia and metabolic dysfunction. 2023;14:391-405.

S20. Lortie J, Rush B, Osterbauer K, Colgan T, Tamada D, Garlapati S, et al. Myosteatosis as a shared biomarker for sarcopenia and cachexia using MRI and ultrasound. 2022;3:896114.

S21. Lorenzo C, Haffner SM, Stančáková A, Kuusisto J, Laakso MJTJoCE, Metabolism. Fasting and OGTT-derived measures of insulin resistance as compared with the euglycemic-hyperinsulinemic clamp in nondiabetic Finnish offspring of type 2 diabetic individuals. 2015;100:544-50.
